# "Carrying Ibuprofen in the Bag": Priority Health Concerns of Latin American Migrants in Spain- A Participatory Qualitative Study

**DOI:** 10.1371/journal.pone.0136315

**Published:** 2015-08-28

**Authors:** Maria Roura, Federico Bisoffi, Barbara Navaza, Robert Pool

**Affiliations:** 1 ISGlobal, Barcelona Centre for International Health Research (CRESIB) Hospital Clínic, Universitat de Barcelona, Barcelona, Spain; 2 Centre for Social Science and Global Health University of Amsterdam, Amsterdam, The Netherlands; UNC School of Dentistry, University of North Carolina-Chapel Hill, UNITED STATES

## Abstract

**Background:**

An estimated 2.7 million Latin Americans reside in Europe, mostly in Spain. Part of a broader project aimed at developing a research agenda on the health status and determinants of this population, this qualitative study engaged Latin American migrants in the identification of research priorities.

**Methods:**

We conducted 30 group discussions between November 2012—March 2013 with 84 participants purposively selected for maximum diversity in Madrid and Barcelona (Spain). We facilitated sequences of task-oriented visual activities to explore their views on priority health concerns. We tape-recorded and transcribed discussions and developed a coding frame based on socio-ecological frameworks, which we applied to all the data using NVIVO-10. A final round of eight group discussions allowed us to triangulate and enrich interpretations by including participants’ insights.

**Findings:**

The cumulative toll of daily stresses was the major health concern perceived by a population that conceptualised ill-health as a constellation of symptoms rather than as specific diseases. Work-related factors, legislative frameworks regulating citizenship entitlements and feeling ethnically discriminated were major sources of psycho-social strain. Except for sexually transmitted infections, participants rarely referred to communicable diseases as a concern. The perception that clinicians systematically prescribed painkillers discouraged health seeking and fostered self-medication. Participants felt that the medicalised, chemicalised, sexually liberal and accelerated culture of the host society damaged their own, and the local populations’ health.

**Conclusion:**

Health systems bear a disproportionate responsibility in addressing health problems rooted in other sectors. Occupational and migration policies should be recognised explicitly as health policies. The mismatch between researchers’ emphasis on communicable infections and the health concerns of Latin American migrants highlights the need for greater interaction between different forms of knowledge. In this process, the biomedical culture of reliance on pharmacological solutions should not remain unquestioned.

## Introduction

The continuous political and economic instability in many Latin American countries and the United States’ tightening of migration conditions and controls have positioned the European Union (EU) as an alternative destination for Latin American migrants (LAM). Spain in particular experienced massive migration from Latin America (LA) between 2000 and 2006, although other countries like Italy and Portugal also saw a considerable increase in their populations of Latin American origin. This relatively new migrant group is composed mainly of Ecuadorians, Colombians, Peruvians, Bolivians and Brazilians who often work in low-paid sectors where irregular employment is common, such as services, construction, domestic work and personal care.

The recent economic recession and high unemployment rates in South-European countries have caused secondary migration flows back to the countries of origin, the Unites States and other European countries [1]. Nonetheless, with an estimated 1.6 million resident Latin Americans, Spain continues to be the most important reception country for this population within the EU. LAM benefit from *positive selection* legislation that facilitates the acquisition of Spanish nationality after two years of continued residence. Still irregular foreign residents from Latin America were estimated at 478,000 in 2008 [2].

Undocumented migrants in Spain can be detained and retained for up to 60 days. Violations of human rights at custody centres [3] and *race-based* police raids aimed at identifying irregular migrants in public spaces such as metro stations and *Internet cafés* have been denounced by a number of organizations [4]. In 2013, a total of 23,889 irregular migrants were repatriated [5].

As part of the austerity measures that accompanied the economic recession in Spain, a Royal decree-law came into force in 2012, which excluded undocumented migrants from full healthcare entitlements [6, 7]. The *congestion* of emergency services caused by this policy recently led authorities to announce partial reinstitutions of their health care rights [8].

Although migrants tend to be, at least initially, relatively healthy compared with the non-migrant population in the host country, they are often more vulnerable to communicable diseases, occupational health hazards, injuries, and maternal and child health problems [9]. Many are exposed to social, occupational and economic conditions that may have detrimental effects on their health [10]. Research on the health of LAM in Europe has focused mostly on *communicable infections*, a range of *psycho-pathologies* and *maternal and child health*, with a predominance of studies focused on *Chagas disease*, a potentially life-threatening vector-borne disease that is particularly prevalent amongst migrants originating from parts of Bolivia [11]. LAM have a tendency to be overweight and are prone to allergies and asthma. Specific sub-populations carry a disproportionate burden of communicable infections such as HIV and tuberculosis (TB). Inconsistent results cast doubts on some widespread assumptions, including popular perceptions that LAM are more prone to psychological disorders and resort to health care services more frequently than the indigenous population. The role played by perceived discrimination has also been insufficiently explored, with studies finding both strong and no association with mental health indicators [1].

Providing an adequate response to the health needs of migrant populations is imperative on both human rights and public heath grounds but it is impeded by major cultural, legal, administrative, political and financial challenges. While the importance of *participation* by intended beneficiaries in the planning of health services is recognised as key to responding to some of these challenges [12–14], there have been few attempts to engage migrant populations in the definition of priorities [15]. Discussions often take place among experts *over the heads* of migrant populations and priorities are generally driven by the interests of academics, policy makers and clinicians [12].

This study is part of a broader project which aimed at developing a multi-disciplinary research agenda on the priority health issues that affect LAM (*Coordinating Resources to Assess and Improve the Health of Latin American Migrants in Europe-* COHEMI). Based on the assumption that the health priorities of migrant populations are not necessarily aligned with those of scientists and that lay opinions constitute a valid source of knowledge, this qualitative study challenged traditional hierarchies of control over knowledge production by engaging LAM in the development of the research agenda, and giving them a voice in the identification of priorities right from the onset of the process.

## Methods

We conducted 30 group activities with 84 persons from 14 Latin American countries in the two cities hosting the largest numbers of LAM in the EU (Barcelona and Madrid). In cooperation with community-based organisations we recruited participants purposively to maximize diversity of ages, nationalities, duration of stay, family circumstances, sexual orientation, and economic, occupational and educational background. We worked with already existing *natural groups* that gather regularly to play sports, practice folkloric dances or pray, as well as vulnerable and/or harder-to-reach persons such as participants in a voluntary return program, women without any formal education, young mothers, sex workers, and sexual minorities, including men who have sex with men (MSM) and transgender women. We considered as Latin American migrants all persons currently residing in Spain that were born in any Spanish or Portuguese speaking country located in Central-South America and the Caribbean. We pragmatically included their descendants in our definition in order to account for potential generational divergences. The majority of study participants had lived in Spain for more than seven years, 85% originated from urban areas, and most had secondary or university education. Further details on the characteristics of study participants are provided in Table 1.

**Table 1:** Characteristics of study participants.

Group	Location	Age range	Country of origin	Male	Female
1. Folklore dance group	Barcelona	20–45	Bolivia	9	9
2. Participants in return program	Barcelona	20–55	Colombia	0	3
3. MSM/transsexual	Barcelona	25–55	Various countries	4	8 (trans)
4. Sport club	Barcelona	25–45	Ecuador	5	0
5. Adventists church	Barcelona	30–55	Various countries	6	6
6. Cleaners	Madrid	25–55	Peru, Ecuador	0	5
7. Adolescents	Madrid	18–25	Ecuador	2	2
8. Illiterate women	Madrid	25–55	Various countries	0	11
9. Young mothers	Madrid	18–25	Ecuador, Colombia	0	2
10. Sex workers	Madrid	35–45	Various countries	0	4+2 (trans)
11. MSM/transsexual	Madrid	25–55	Various countries	3	3 (trans)
**TOTAL**				**29**	**55**

We gathered a total of 11 different groups and conducted repeated discussion sessions with each of them at their usual gathering venues or in rooms facilitated by community-based organisations. We aimed at organising three rounds of activities with each group in order to engage participants in the identification of priority health concerns (Round 1) and possible solutions (Round 2) using task-focused activities specifically tailored to encourage groups to work together in the identification, ranking and analysis of their most prominent health concerns. During these activities, participants were asked by the facilitators (one anthropologist and one cultural mediator trained in qualitative methods) to identify main health problems using symbolic *bricks* that were ranked by participants into a *wall of challenges* that the researchers used to probe further and gather additional insights. The use of open-ended questions gave participants the opportunity to respond in their own words as opposed to forcing them to choose from fixed responses [16]. During Round 2, the researchers catalysed a deliberative process aimed at co-creating a *tree of solutions* where symbolic *fruits* represented potential ways to overcome the challenges identified during the previous round. The initial data collection guideline used during these activities is provided as supporting file 1 (S1 File). We encouraged participants to contribute to the interpretation of our findings by organising a third round of discussions where we shared our study results and engaged them in the refinement and revision of our interpretations (Round 3).

A generous time allocation of approximately three hours per activity allowed us to progressively build trust and gain depth of insight and analysis so we could explore emerging topics, share our findings with participants and validate our interpretations in a two-way communication flow between social scientists and study participants. We brought together 65% of participants from eight of the groups for three consecutive rounds and a total of nine new persons joined us for Round 2 or Round 3. For three of the groups it was not possible to organise a final session due to difficulties in convening the group within the time frame of our study. Overall, 42% of study participants took part in the three rounds (Table 2).

**Table 2:** Number of participants by group and data collection round

	Round 1	Round 2	Round 3
Group 1	11	10	0
Group 2	3	3	0
Group 3	12	7	4
Group 4	5	4	3
Group 5	12	6	0
Group 6	5	3	3
Group 7	3	4	4
Group 8	11	9	11
Group 9	2	2	2
Group 10	5	7	2
Group 11	6	3	3
**TOTAL**	75	58	32

Data collection, processing and analysis took place simultaneously allowing for timely adaptation of data collection guidelines as soon as salient new topics were identified [17]. During the fieldwork period (November 2012—March 2013) we organised weekly debriefing meetings to adapt the tools based on the contributions of a multidisciplinary team composed of a sociologist, two anthropologists and a cultural mediator. New questions and probes were added to the initial data collection guidelines as a result of this ongoing process.

All discussions were tape-recorded and transcribed (BN, FB). We also took notes and pictures of visual outputs. The quality of transcriptions was checked and the process fully revised when deemed necessary. The first author (MR) conducted the analysis of data in its original language (Spanish) with inputs from FB and BN. Recurrent topics were assigned codes and were sorted into categories and subcategories within an initial coding frame that accounted for the diverse layers of health determinants underscored by the socio-ecological theoretical framework developed by Dahlgren and Whitehead including the broader structural context, living and working conditions, social networks and individual factors [18]. Codes continued to be created, merged and modified as data collection and analysis progressed, employing a combination of inductive and deductive approaches. A definitive coding framework was agreed on through research team consensus and then the first author coded all the data with the aid of NVIVO-10 software [19, 20]. We matched our findings against the key areas of research identified in a recent systematic literature review focused on LAMs’ health [1] in order to identity most prominent gaps between the current scientific focus and participants’ views on what the priorities should be. We paid particular attention to disconfirming evidence in order to provide a nuanced account of our findings and refine emerging interpretations [17]. During the third round of activities we presented our preliminary results and collected participants’ views on our preliminary findings. One of the facilitators (BN) ensured that the doubts raised and gaps in knowledge identified were clarified for participants. She distributed informative leaflets and telephone contacts where participants could obtain further information including medical, social and legal advice.

Ethical approval was obtained from the Hospital Clinic-University of Barcelona ethical committee board (CEIC). Informed consent procedures were administered throughout fieldwork. At the recruitment stage all prospective participants were informed in detail about the overall aim of the project, the approach used and their expected role. We highlighted the voluntary nature of participation and asked participants for consent to be anonymously quoted in study reports so they could take a decision about whether or not to attend the activity. In addition, prior to each group discussion, an information sheet was read-out and sufficient time allocated for questions. Special emphasis was placed on avoiding false expectations and clarifying the boundaries of the potential support to be offered by the project. All necessary information was provided in a language that suited the participants' level of understanding and the group facilitator stressed that each participant was free to leave the group at any time or to refuse to participate in any of the activities. As this study carried a minimal risk, attendance and participation in the activities was considered sufficient proof of consent by the ethical committee board. No remuneration or compensation was provided to participants, except for a small lunch shared with the researchers during the breaks in the activities.

## Results

The main health concerns identified by study participants were consistent across groups and related to stressful working and living conditions associated with their condition as migrants and further exacerbated by the *accelerated rhythm of life* in the host society. Participants consistently delineated a body-mind continuum where emotional and health problems were intrinsically linked and manifested in a constellation of ill-health symptoms rather than as specific diseases.

### Occupational stress: “working yourself to death”

Occupational strains were perceived as a critical determinant of health in all the discussions. Having adopted a work-centred life, ill-health was a severe impediment to attaining the objectives of a predominantly economically-driven migration project.

F4: We work ourselves to death and forget about ourselves. If I want to buy something I have to work and keep on working. We kill ourselves bit by bit and we do not realise until those ailments appear.

Group 1, round 1-Bolivia

Lack of sick and maternity leave entitlements were also mentioned as major concerns in a population that often worked under irregular conditions (“we all work in the black economy, all of us”). Excessive work demands, job insecurity, unemployment and low status jobs were commonly reported sources of stress. Unmet expectations based on assumed congruity with the *Spanish culture* were similarly raised as causes of poor health.

F2: To work is more tiring here than in our home countries because here we must do everything quickly, within strict time limits. It feels like time goes by faster and you get more tired.

F3: It is the socio-cultural atmosphere. The culture here overwhelms us, the excessive workload.

Group 2, round 1- Colombia

M1: You arrive here expecting to have a better life and then you find out that you have to wake up at 5 am in the morning and start cleaning toilets even if you have a good CV. That’s what causes stress and all the symptoms.

Group 3, round 1- various countries

### Irregular residence status and discrimination: “sudacas” and “panchitos”

Several participants mentioned that ethnic discrimination affected their health because they were physically recognised as *panchitos*, *incas*, *indios* or *sudacas* (terms applied derogatively to LAM in Spain).

F1: Here I learnt a horrible word: *sudaca*. It affects you to be called like that…What? Me? *sudaca*?.

Group 2, round 1- Colombia

M6: They see your South-American face: a shitty *sudaca* who came here to deal drugs, rob and do indecent things.

Group 1, round 2- Bolivia

For some, the economic recession had increased discriminatory attitudes. The legislative environment was seen as a crucial determinant of health for migrants with irregular residence status as they lived in constant fear of being arrested and/or deported.

M3: We live in uncertainty and feel insecure. When an undocumented person walks by the street s/he is nervous. S/he is not a delinquent but being undocumented is a fault for the police.

Group 11, round 1- various countries

### An accelerated rhythm of life: “catching stress”

Participants described the overarching accelerated *rhythm of life* prevailing in the reception society as a major source of stress, which was seen as *contagious* and could be “caught”.

F2: I started to walk fast because here everybody walks fast. I saw everyone getting out of the metro in a rush and I became accelerated as well […] when I arrived here I almost killed myself trying to catch up with the rhythm of Spanish people. In the metro, the door opens and everyone gets out like cattle and it is contagious! You get used to it. Here everything is under pressure. Everyone is hurried. I am always running. I caught it! I’ve ended up doing everything mechanically.

Group 10, round 2- various countries

In this context, the cumulative succession of challenges faced in their daily lives caused ill-health through a reduction of *body defences* and adoption of unhealthy behaviours. Social support could help to cope with emotional downturns but many participants felt ultimately alone in the face of day-to-day problems.

F1: Everything happens at the same time. It is like being on the front line of the battle, and you have to work out all those things, one after another, very fast. Then the immune system goes down. It gets depressed.

Group 11, round 1- various countries

F2: you carry it [stress] with you. You wake up with it and you go to bed with it

F8: we are always looking at the watch, worried about being on time

M11: And coffee, a lot of it, to be awake, to work better, because you are so tired.

Group 1, round 2- Bolivia

Stress was manifested in anxiety, insomnia and depression, and was associated with deterioration of the immune system and with a constellation of symptoms often characterised by pains (backache, headache, migraine, stomach ache, and bone pain). Backache in particular ranked high as a priority concern in almost all the activities held. Within a vicious circle, participants perceived that stress could cause more serious diseases such as cancer, which would synergistically translate into higher levels of stress and poorer health.

Depression was mentioned by the majority of groups as a priority health concern and it was generally attributed to excessive work load, being separated from family, low status jobs, and unemployment. The interpretation of symptoms commonly ascribed to depression such as sadness and apathy varied widely. For several participants, these were *normal* emotional responses to harsh living and working conditions but one participant explained vividly how her symptoms *perfectly matched* with the medically defined syndrome known as *migratory grief*. The existence of a diagnostic category under which her distress could be labelled was comforting for this woman: it provided her with a *secure* diagnosis and legitimated her status as *sick*, helping her to *make sense* of her symptoms.

F1: During my first year here I just wanted to cry. I felt lonely. I searched on the Internet and I found out that this has a name: *migratory grief*. I read the symptoms, and that is exactly what we feel when we arrive. I felt very sensitive, anything made me cry. That happens because you are going through and adaptation process that is called *migratory grief*. It happens to all of us but many people don’t know that it has a name. They think it is an emotional downturn but it is not. I did not invent it. There is a report about it that you can find on the Internet. It includes all the symptoms. We are all suffering from it but many are not aware of what is happening.

Group 2, round 1- Colombia

A few participants said that *depression* did not exist as a disease in their home countries; others believed that although it existed, people did not recognise it as such. When we brought our preliminary results back to participants, one woman vividly questioned the pre-eminence of *depression* as a major health priority in our findings.

F2: The importance of *depression* is overstated in your results. I learnt about that disease here. In my country it is not well known. I don’t think it is actually a disease. In my country nobody goes to a psychologist. We are used to be strong and brave. We can catch it here where being depressed seems to be a normal thing but the truth is that… you [directing the question to another participant]: How many depressed friends do you have? And you [directing the question to another participant]: How many depressed friends do you have? And you [directing the question to another participant]: How many depressed friends do you have? [no answers]. I haven’t got a single one! You go to the *Latino* disco and we are dancing every Saturday. You go to the parks and it is full of us [laughter from other participants]. I believe that in our social environment, *depression*?. [sceptical tone]

Group 6, round 3—Peru and Ecuador

### Health behaviours: “eat, eat, and eat, because of anxiety”

For most study participants *stress* was closely associated with unhealthy behaviours. Many reported an increase in dietary fat intake after migration, which was generally attributed to anxiety and lack of time to cook. Less frequently, stress and economic problems were also associated with weight loss.

F2: Everyone who comes here gets fatter. I have gained ten kilos since I arrived. I think this is caused by the anxiety of being in a different environment […] You eat and eat and eat, because of anxiety. It all begins with stress, anxiety, eating disorders, worries. Psychological stress always leads to physical problems. The brain is a very powerful weapon.

Group 2, round 2- Colombia

Both males and females mentioned unhealthy behaviours such as excessive eating, and alcohol, cigarette and illicit drug consumption as ways to *escape from problems*. The greater social acceptability of cigarettes in Spain could foster smoking habits in females.

M2: Tobacco, above all tobacco. There are women who did not smoke in Ecuador and here they smoke because there is more freedom. Here nobody tells you anything. Instead there in Ecuador, if you smoke, people will talk (gossip). In those circumstances it is embarrassing to hold a cigarette.

Group 4, round 3- Ecuador

### Allergies and skin problems: “from natural to chemical”

Other commonly reported health problems such as allergies, skin problems, pains and colds were attributed to seasonal temperature changes and the use of chemically treated water. Atmospheric contamination and environmental noise were also mentioned as determinants of poor health, especially amongst participants from rural areas.

F4: The water in Spain is contaminated

F3: I come from a part of Peru where we drink water directly from the river, it comes down from the mountain and it is very healthy.

Group 6, round 1- Peru and Ecuador

F2: You switch from something natural to something that has lots of…

M2:… chemicals.

Group 7, round 1- Ecuador

F2: I don’t like the summer at all. I can’t stand the allergy I get during those months. It is a nightmare.

Group 6, round 3- Peru and Ecuador

### Sexually transmitted infections: “there is more promiscuity here”

Participants rarely mentioned specific diseases as a health concern, with the exception of STIs, and particularly HIV, which emerged spontaneously as priority areas to be addressed in discussions held with *high-risk* populations (MSM, transgender females and commercial sex workers), as well as amongst heterosexual males and female domestic workers from Andean countries. Both men and women acknowledged having adopted more *liberal* sexual behaviours and this was mainly attributed to the culture in Spain where sexual ethical codes were perceived to be more relaxed. Feeling lonely, sharing accommodation and drinking alcohol were seen as triggers of sexual activity in women. Interactions with locally-born friends holding more liberal attitudes towards sex fostered what was perceived as a *promiscuous* sex life that contrasted with the more conservative sexual code reported to prevail in the countries of origin. This perception was shared in all the discussion except for the group of youths who perceived that risks in Spain were lower here because they had greater access to information.

F7: We are more conservative because we were raised that way: first you close the legs, and then you get to know the person.

F2: but here it is different. There is never anything stable. Then you get infectious diseases […] one, two, three shags [polvos] as you [Spanish] say.

Group 8, round 1- various countries

### Diseases of the poor: “tuberculosis, lice and Chagas”

Leaving aside STIs, the only infectious disease that was prioritised by more than one group was tuberculosis. Other communicable conditions were mentioned only once, and included Chagas, dengue, fungus, hepatitis, leishmaniasis and lice. Tuberculosis was seen as severe because it was very contagious and potentially lethal, but was not perceived as *a Latin American problem*.

Chagas disease was not identified as a priority health concern even amongst people from Bolivia, where it is highly prevalent. When the facilitator prompted discussion about this disease—probing with popular terms such as *vinchuca*, *chupón* and *barbeiro—*low risk-awareness and knowledge gaps became apparent. Chagas disease was perceived to be a disease of people living in extreme poverty with very poor hygiene. In a group of 12 men from endemic countries, only three knew about it as a disease that was “not happening any more” because “even the indigenous no longer live in tents”. The disease was perceived to be “under control” and ascribed to “careless” persons. A few persons held the misconceived view that they had been vaccinated against it.

F1 (Bolivia): You find it above all in rural areas, where there are animals

F2 (Bolivia): That is why before we arrived we were vaccinated against it

F1 (Bolivia): In my country Chagas is well controlled. If you have it, it is because you have been really careless. You see it only in places where people don’t even have water. That type of disease is well controlled. Chagas, tuberculosis and lice are well controlled

F3 (Honduras): In Honduras there used to be Chagas but they control it well

F2 (Bolivia): Through vaccinations, they control it well

F3 (Honduras): You often find it in prisons

F2: In unhealthy places

Facilitator: and in Colombia?

F4 (Colombia): No, I did not know about Chagas. I was aware of bed bug and lice, but not Chagas.

Group 8, round 3- various countries

### Non-communicable conditions

Cancer, thyroid problems, anaemia, kidney stones, appendicitis and arthritis where mentioned only occasionally. Similarly, cardio-vascular diseases were rarely raised as a health concern. When they were mentioned, they were associated with high cholesterol levels caused by an “abuse of fast food” and a stressful lifestyle caused by long-working hours. Alcohol and tobacco consumption were equally related to stress but not explicitly associated with cardio-vascular pathologies. Cancer was mentioned as a health concern in only two groups, but ranked high as a priority due to its high lethality.

M1: The rhythm of life here, stress, worries…it causes hypertension in the heart.

Group 7, round 1- Ecuador

### The health system: “ibuprofen and paracetamol”

Consistent with perceived priority health needs, health care was commonly sought for unspecific symptoms as opposed to concrete diagnosable diseases.

A range of barriers prevented some persons from accessing health services. These mainly related to recent legislative changes restricting health care entitlements for irregular migrants. Not having a *health card* and fears of being arrested were key obstacles in a context where irregular work was common and confusion over the actual implications of new regulations prevailed. Restrictions of health care entitlements could lead to deployment of a range of strategies to access health care, including borrowing medical cards and resorting to emergency care for non-urgent conditions. Bureaucratic procedures, lack of information, incompatibility of work duties with hospital schedules, and concerns over being diagnosed with a serious disease also hindered service use. In a population unanimously self-defined as stressed, *time* was a highly valued asset that was carefully administered to fit in competing priorities. Earning hourly-paid wages ranked high in the hierarchy of life priorities and was often prioritised over attending a doctor or getting the necessary rest to recover from illness (“You have to choose between your work and your disease”).

Factors influencing health seeking that transcended access barriers included the cost of medicines, not being physically examined and having to go through general practitioners in order to access specialised care. Many participants reported that they were always prescribed the same medicines (ibuprofen, paracetamol and omeprazole), which discouraged them from seeking care and triggered self-medication. The need to endure pain to be able to work fostered routine use of pain-killers/anti-inflammatory drugs that reached levels categorised as *addiction* by a few participants. The widespread routine medicalisation described by study participants was often attributed to the *local culture*, characterised by marked preference for medicines over natural remedies. (Table 3)

F2: I’ve heard people say: “Why should I go to see a doctor if all what I’ll get is paracetamol? I can just buy it myself in a pharmacy”. Many people have stopped going to the doctor because they just give you paracetamol and that is it!

Group 5, round 2- various countries

A few participants ascribed it to clinicians’ tendency to attribute their *pains* to *stress* or categorise them as *psychosomatic* and hence not deserving additional medical examination.

F4: They always end up prescribing omeprazole. [Pretended doctor’s response]: “Ah, you [Hispano-Americans] complain about minor things”.

M7 [Pretended doctor’s response]: “It is stress”. That’s all what they tell you

M4 [Pretended doctor’s response]: “Ibuprofen”

Several voices [Pretended doctor’s responses]: “Paracetamol”, “omeprazole”.

Group 1, round 1- Bolivia

**Table 3:** Medicated lives: “I carry ibuprofen in my bag”.

Quote	Data source
F2: Everybody carries it. My mother, because she works, she must carry her ibuprofen in the bag. Without it she is not herself. If you have to endure a pain, you will always carry ibuprofen in your bag. That is evident.	Group 3, round 3- various countries
F1: It shocked me. Here for everything, for any kind of pain: ibuprofen. I’ve started taking stomach protectors. I had never heard in my life that they existed because in our countries we have a different culture, we use herbs.	Group 2, round 1- Colombia
F4: Anti-inflammatory drugs, as many as you want; pain-killers, as many as you want; tranquilisers, as many as you want. They have never denied me any medicine. I have a bunch of medicines. If I take all of them I can’t even stand up. I have to go to the toilet every 5 minutes because my stomach is upside down. If I am medicated I can’t work, if I am not medicated I can’t work either.	Group 10, round 1- various countries
F2: […] they are addicted to it […] there, where I work, the cleaning ladies. They have backache, they have another pain there… [Pretending colleagues’ voices]: “I’ll take ibuprofen because I can’t bear this backache”, and they always go around asking for ibuprofen. My cousin the same, with paracetamol […] every day I hear her say that she is going to take paracetamol.	Group 6, round 3—Peru and Ecuador

Views on the quality of health-care varied and were mostly influenced by the attitude of professionals. In spite of sharing the Spanish language some participants reported communication problems with providers. These included use of medical jargon and a tone of voice described as *rough*. Some felt discriminated in the attention received but others attributed it to *Spanish culture* and sympathised with the deteriorating working conditions of health professionals. The functioning of the health system compared favourably with services received in the home countries, the free services were appreciated, and the system was seen as particularly efficient in dealing with severe diseases such as cancer and HIV.

When we asked participants to identify interventions that would benefit their health, most proposals targeted health institutions and providers. These included training HPs on interpersonal relations, the establishment of monitoring mechanisms to identify discriminatory practice, the diversification of clinicians’ ethnic profiles, expanded presence of cultural mediators at health facilities, and provision of services beyond usual working hours. Participants also mentioned that they would benefit from psychological support services, provision of clear information about their entitlements and occupational safety courses specifically adapted to their needs (E.g. ergonomic advice for cleaners and carers). They mentioned that they should take care of themselves (“eat well, sleep well”) and that *veteran migrants* could be involved as mentors providing health support and advice to new comers.

## Discussion

This qualitative study found that *stress*, and to a lesser extent *depression*, were the major health concerns of a population that conceptualised their health status as a constellation of symptoms, often manifested as *pain*, rather than as specific diseases. Participants described a generalised situation of stress that could trigger the adoption of unhealthy habits, which they attributed to the cumulative toll of challenges they had to face on a daily basis, with a particular predominance of work-related factors. The accelerated rhythm of life prevailing in the reception society was felt to be *contagious* and to contribute to the high levels of stress reported by study participants. Other sources of chronic strain related to the legislative framework regulating citizenship entitlements and feeling ethnically discriminated. Colds and other ailments attributed to environmental conditions were also frequently mentioned, with episodes of asthma and allergies commonly reported. With the exception of STIs, participants rarely mentioned specific diseases as a concern. Health seeking could be hindered by lack of access to services, short-term prioritization of work over health, and the perception that they were always prescribed the same drugs: ibuprofen, paracetamol and omeprazole (Table 3). During a final round of group activities designed to engage study participants in the interpretation of results, stress was ratified as the leading overarching perceived cause of ill-health, but the conceptualization of *depression* as a disease was sometimes questioned.

The body-mind continuum consistently described by participants and the relevance attributed to relatively common ailments such as anxiety, migraines, colds, digestive problems, and backache, is in sharp contrast to the vertical, disease-centred approach that prevails in the scientific literature addressing LAM’s health in Europe [1]. In our study, participants of both sexes acknowledged having adopted more liberal sexual behaviours after migration, and only the youngest reported adoption of safer-sex practices, which they attributed to increased availability of information. Most were unaware of risks related to diseases known to be particularly prevalent amongst specific Latin American populations including TB and Chagas disease [1, 11]. Our findings thus emphasise the importance of paying increased research and policy attention to communicable diseases that disproportionately affect LAM. In addition, it should be acknowledged that communicable diseases contribute only partially to the burden of ill-health in a population that is more commonly affected by gynaecological and obstetric conditions, traumatic accidents, migraine, headaches, lower back pain, anaemia, emotional downturns and gastrointestinal disorders [1]. Our results are in line with studies conducted in other populations, which also suggest that migrants are commonly affected by the same diseases as locals [12, 21–23]. The few conditions for which migrants have specific health needs must be addressed but the focus on differences rather than similarities should not divert attention from the most common causes of morbidity in this, and other migrant populations.

For our study participants, job insecurity, unemployment, long working hours, communication problems with employers and low status employment were important sources of stress and major underlying reasons for most of the symptoms described. Within a perceived vicious circle, body defences deteriorated and unhealthy mechanisms to cope with stress were deployed, including anxiety-induced unhealthy eating, smoking and alcohol abuse.

The aetiology of disease described by our study participants resembles the accounts provided by social epidemiologists on the multiple pathways through which the psycho-social environment influences health outcomes, both directly through deterioration of the immune system and indirectly via adoption of unhealthy behaviours [24]. The brain is able to influence immune function [25] and a growing body of research has examined the pathways though which subjective experience and emotions translate into chronic stress to affect health outcomes [25, 26]. As described by our study participants, these involve the immune system, but also metabolic and neuro-endocrine reactions [25].

Psychobiological studies suggest that while the stress response is an essential element in the total adaptive system of the body, a period of recovery is necessary to manage new demands [27]. Our study participants vividly described feeling overwhelmed by a cumulative succession of daily challenges, suggesting that a period of recovery was often unavailable to them. This *allostatic load* associated to repeated or chronic challenges [28] can potentially lead to sustained psychobiological activation and loss of dynamic capacity to respond to new challenges, and has been related to higher risk of illness among people with lower socio-economic status [27]. Additional research is needed to assess how the potential accumulation of psychosocial disadvantage may exacerbate these processes in this population and the potential effects on health of the coping/resistance strategies mobilised [29].

The participants’ view that they were *working themselves to death* is consistent with epidemiological research pointing to occupational strain as a major cause of ill-health [30, 31]. High job strains and effort-reward imbalances [32, 33], limited decision-making autonomy [34, 35], shift-work [36], job insecurity [37], unemployment [38] and lack of opportunities to use skills [30] have been reported as detrimental to health in the general population. These factors may be exacerbated in migrant populations that are less likely to have job security, more likely to work at a very high speed, and to work shifts or at least have variable working hours [39, 40]. Our study participants reported common effort-reward unbalances including employment in low status jobs, occupational insecurity, and poor promotional prospects. Qualitative research should build on sociological theories commonly used in epidemiological research—such as the *effort-reward* imbalance model [33]—to examine its applicability to diverse migrant populations. The degree to which strains and/or buffers rooted in other spheres of life (emotional, relational, financial) synergistically operate to negatively and/or positively influence migrants’ health status and behaviours also deserves further attention. Greater policy attention should be paid to the *invisibility* of domestic and personal care work frequently performed by LAM [41]. A careful evaluation of the achievements and pitfalls of policies designed to regularise domestic work, such as the voucher system in Belgium [42], could shed light into what should be done to address this issue.

Unhealthy behaviours such as smoking, excessive eating and alcohol/substance use were generally seen as mechanisms for coping with stress. This highlights the importance of addressing the underlying determinants of behaviours [43], moving beyond bio-behavioural reductionist health promotion interventions that subtly blame the affected ones out of their *ignorance* and/or *unwillingness to change*. To transcend the current narrow focus on *culturally friendly* information materials we need to further understand the linkages between contextual factors and the adoption of specific health behaviours without neglecting individuals’ personal agency. Inductive theory-generating qualitative inquiries could provide a better account of how self-efficacy, coping mechanisms and resistance/resilience strategies are deployed by populations constrained by major structural barriers, contributing to advance our understanding of the interplay between culture and social structures, and migrants’ possibilities to influence their lives [44].

The health problems prioritised by the participants in this study were strikingly consistent across heterogeneous groups, and participants often referred to certain characteristics of the host society as sources of ill-health. These were shared with the local populations and included an *accelerated rhythm of life*, greater exposure to chemical products, more *liberal* sexual norms, social acceptability of some unhealthy habits and widespread medicalisation. The health problems associated with stress were not necessarily seen as specific to migrants or LAM but rather as a particularity of the receiving country where stress was part of *normal life* and could be “caught”. Still, many study participants reported that they were also affected by sources of stress that were specific to their status as migrants, and which derived from discrimination, irregular residence status, occupational disadvantages, financial concerns and poor social support.

Participants were generally satisfied with the health services but a common complaint was that they were always prescribed the same drugs: paracetamol, ibuprofen and omeprazole. This could hinder health-seeking and foster self-medication as a routine strategy to palliate a range of pains (*dolores*), especially when these interfered with their capability to work. While the use of these drugs is widespread in many parts of the world and some participants viewed them as harmless, they are not exempt from pharmacological debate [45–47]. A recent systematic review and a placebo-controlled trial, for example, have questioned the innocuousness of paracetamol [48] and its efficacy for back pain [49].

**Fig 1:**
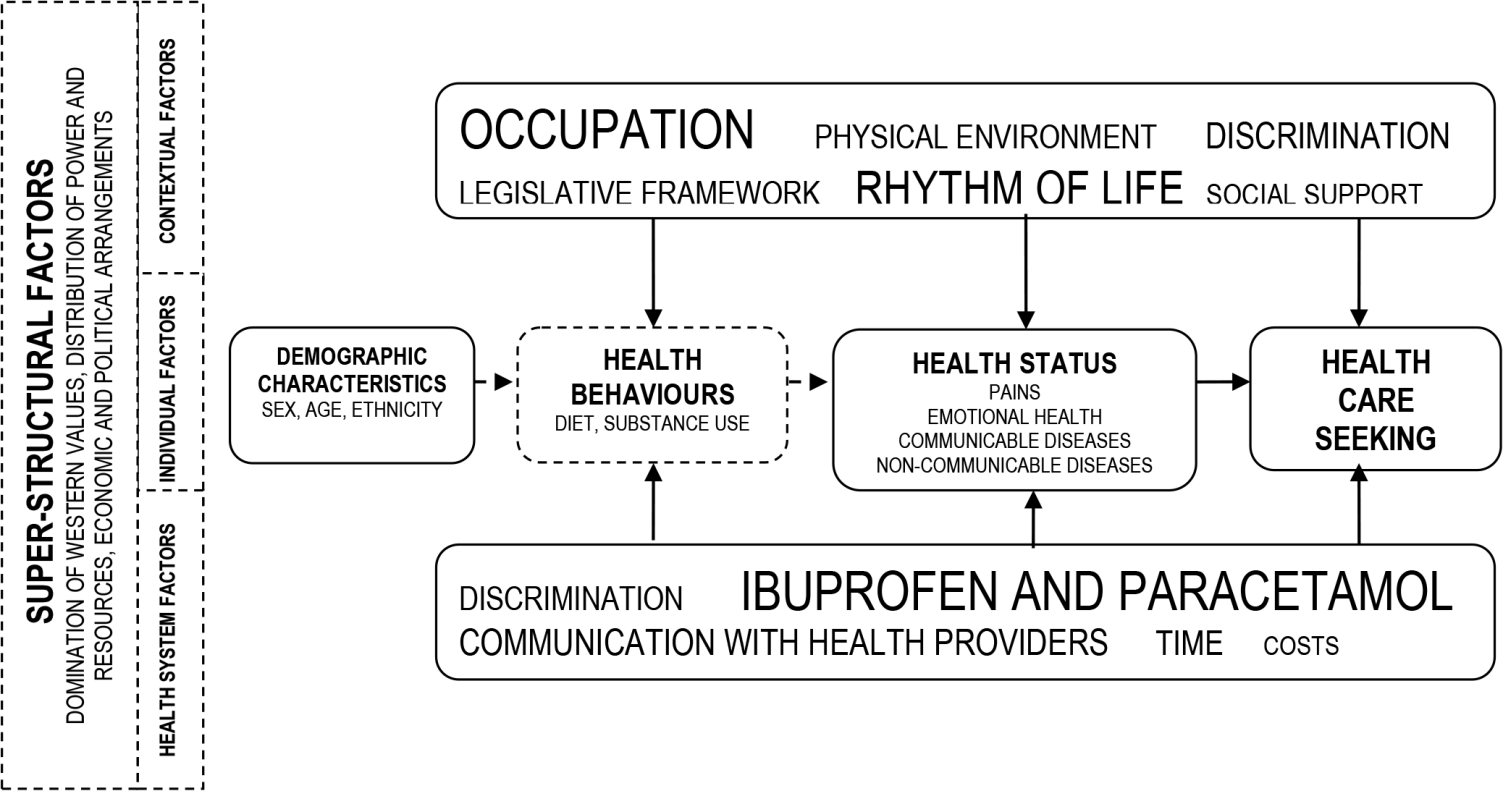
Priority health concerns and determinants identified by study participants.

In contrast with the traditional view of migrants’ culture as a *source of dysfunction*, our results support calls for a more comprehensive approach where *cultures of all kinds*, including professional and political cultures, are tackled as major barriers to better health [50]. The conditions of daily life affect individuals’ health and these are in turn influenced by structural factors including laws and policy frameworks [43] as well as by super-structural determinants related to the hegemony of western values, unequal distribution of power and resources, and macro-social, economic and political arrangements (Fig 1). Beyond the micro-level focus on health behaviours and social networks due attention must be paid to broader structural factors [44, 51], including the underlying reasons of the *accelerated rhythm of life* prevailing in the host society, the medicalisation of common conditions [52] and the socially un-sanctioned practice of employing LAM as domestic workers in irregular conditions. As illustrated by the reports of participants rushing out of the metro “like Colombian cattle”, holding “embarrassing” cigarettes, or having “two, three shags”, behaviours may change faster than values. The emotional unrest derived from this and its implication for health and wellbeing requires additional research.

Quantitative reports point to wide disparities in mental health status across different LAM populations [53] supporting the idea that perceptions of psychological wellbeing differ markedly both across and within societies [50]. Studies examining the mental health of LAM in Europe show inconclusive results [1] and a meta-analysis found that the overall prevalence of depression in migrants is similar to figures for the general population [54]. Still, LAM are frequently portrayed in the popular media and in medical/academic circles as a population that has a low tolerance of pain [55], is prone to depression [56] and somatisation [57], and at high risk of developing psychiatric problems [58]. It could be hypothesised that the *culture* of viewing LAM in this light might have contributed to the over-attribution of their symptoms to a depressed mood. Our data does not allow us to confirm this hypothesis, although concerns about the *pathologisation* of migration have been previously raised. In the UK for example, the higher prevalence of diagnosed mental illness in Afro-Caribbeans is generally attributed to the stressful conditions of migration, but it has been argued that this could also be due to the practitioners’ shared view that this group is particularly prone to mental disorders [50].

Migrants’ cultures (like the host cultures) are hybrid, heterogeneous, many-layered and constantly changing [12, 51, 59]. They draw on many cultural sources through social interactions including encounters with health providers from whom they pick up new schemas through which to think about and conceptualise illness. Health providers participate in a process of mutual interaction that shapes migrants’ culture, influencing their mindset [60]. We can’t rule out that these interactions might have influenced our study participants’ perceptions of their own health, leading to frequent prioritisation of *depression* as a health concern. If, as suggested by medical anthropologists, the differences in the cultural meaning of depression alter individuals’ experience of depressed mood [61], the categorization as *disease* of what could also be considered as a normal pattern of distress in response to stressful life events [50, 62] could negatively influence individuals’ self-perception of wellbeing [60]. Tagging a group with a diagnostic label is problematic as once integrated into a set of *cultural beliefs*, labels are difficult to remove and may be internalised by individuals (and groups) who accept the label they have been assigned, potentially reinforcing the expectations created by that label. It should also be considered that cultural variations in the way distress is experienced and expressed may lead to under-diagnosis of mental health problems because diagnostic tools based on western expression of emotional wellbeing may be less sensitive in specific migrant groups who also may experience poorer access to medication and care [63]. It is important to consider the potential harm resulting from unnecessary labelling [64] attached to shifting medically-defined boundaries between the *norma*l and the *pathological* [52]. At the same time, it should be acknowledged that the construction of a diagnostic term may help some persons to *make sense* of their suffering, legitimate access to services and establish entitlement to rights reserved for *the sick*. The existence of *mild* categories of mental health problems could foster health-seeking for otherwise highly stigmatised psychiatric conditions likely to remain undiagnosed.

The dramatic discrepancy between the priority health problems addressed in the scientific literature (mostly focused on communicable infections) and the views of LAM themselves (mainly centred around constellations of ill-health symptoms) highlights the need for greater interaction between different forms of expertise. As pointed out in the *Review of Social Determinants of Health* “effective mechanisms are needed to enable individuals and groups who are the target of policy to be heard and meaningfully involved in decisions that affect their lives” [43]. In this process, the *biomedical culture* and its reliance on pharmacological solutions should not remain unquestioned [60]. In our study, participants’ *beliefs* coincided with what has so far been reported in epidemiological research, while health providers often prescribed painkillers of questionable effectiveness in treating common ailments. Our findings cast doubt on the popular assumption that health providers’ *knowledge* is the only valid one, and that concessions must be made to accommodate migrants’ *cultural beliefs* [50]. The adoption of *culturally humble* approaches that question the adequacy of dominant cultural frameworks and foster self-reflection [60] is a potential promising avenue for a “better recognition among those who care for the sick of their own cultural assumptions and biases” [50]. Along the same line of argument, the explicit recognition of the harms of over-diagnosis and overtreatment in some medical circles [64–66] and severe criticism of proliferating diagnostic categories “dressed up by modern psychiatry as diseases” [62] calls for revisiting the assumption that the *biomedical culture* is homogeneous and fixed.

In our study, participants from populations severely affected by Chagas disease—a potentially deadly condition- did not identify it as a major health priority, illustrating the importance of balancing patient involvement with professional medical opinion. While most health problems were attributed to the overarching social determinants of health, the solutions proposed focused on the health sector and disregarded broader level interventions as unrealistic. Important health issues must be addressed even if they are not socially constructed in a community as warranting research and action [67].

This study has several limitations. We did not reach data saturation. The iterative process of collecting and analysing data and feeding results back to participants could have continued to add more depth to our analysis and generate additional insights. The views of persons more severely depressed, isolated or overwhelmingly stressed are likely to have been under-documented as they are probably less likely take part in time-consuming and demanding research. We achieved modest success in sustaining participant involvement throughout the project and acknowledge the enormous challenges laying ahead for our study results to be translated into a policy effectively addressing the broader, macro-structural external layers of the social-ecology. Additional time and resources would have been required to engage relevant parties such as health providers and policy makers to increase the chances of translation of results into interventions. While our findings make a convincing case for the importance of integrating health policies into other sectors, placing health-related goals on the agendas of policy-makers who have other aims and priorities is beyond the scope of this study. In spite of these limitations, we expect that our findings will contribute to stretch the limits of migrant health policies beyond their current focus on infectious diseases and individual lifestyle and will nurture critical thinking about the use of participatory approaches in health research. As the importance of accounting for diverse forms of knowledge is increasingly recognized in scientific circles [68] and the *community engagement* paradigm is growingly embraced in health research beyond its traditional focus on HIV [69–70], it its crucial to account for the *lessons learnt* and acknowledge the challenges posed, resources needed, and limitations of this increasingly popular approach [71, 72].

## Conclusion

It is important to tackle both the diseases that *disproportionately* affect LAM and the health conditions that affect them *more frequently*. Health systems face limitations in addressing health problems whose roots are embedded in broader contextual factors. Occupational and migration policies should be tackled as major structural determinants of health. The consideration of migrants’ strengths, vulnerabilities, commonalities, heterogeneities and continually re-constructed *hybrid cultures* adds layers of intricacy to a field of undeniable empirical complexity.

The mismatch between researchers’ emphasis on communicable infections and the health concerns of Latin American migrants highlights the need for greater interaction between different forms of knowledge. In this process, the biomedical culture of reliance on pharmacological solutions should not remain unquestioned. The limitations and costs of participatory approaches must be acknowledged.

## Supporting Information

S1 File Data collection guidelines. (PDF)

## References

[1] Roura M, Domingo A, Leyva-Moral JM, Pool R. Hispano-Americans in Europe: what do we know about their health status and determinants? A scoping review. BMC public health. 2015;15(1):472 . Pubmed Central PMCID: 4430018. 25948239 10.1186/s12889-015-1799-x PMC4430018

[2] International Centre for Migration Policy Development. Clandestino Database on Irregular Migration. Stocks of Irregular Migrants: Estimates for Spain 2012. Available: http://irregular-migration.net/typo3_upload/groups/31/3.Database_on_IrregMig/3.2.Stock_Tables/Spain_Estimates_IrregularMigration_Nov09_2.pdf.

[3] Campanya Tanquem els CIE. Campanya pel tancament dels CIEs 2015. Available: http://tanquemelscies.blogspot.com.es/p/qui-som.html.

[4] Ceberio M. Interior prohíbe las redadas indiscriminadas de inmigrantes. El PAIS. 2012. Available: http://politica.elpais.com/politica/2012/05/20/actualidad/1337496910_464121.html.

[5] Ministerio de Interior, Gobierno de España. Lucha contra la inmigración irregular—Balance 2013. 2013. Available: http://www.interior.gob.es/documents/10180/1207668/balance_2013_inmigracion_irregular.pdf/132387b3-d93b-4485-8a5b-1a734359764c.

[6] Legido-Quigley H, Otero L, la Parra D, Alvarez-Dardet C, Martin-Moreno JM, McKee M. Will austerity cuts dismantle the Spanish healthcare system? Bmj. 2013;346:f2363 10.1136/bmj.f2363 23766463

[7] Casino G. Spanish health cuts could create "humanitarian problem". Lancet. 2012 5 12;379(9828):1777 . 22586719 10.1016/s0140-6736(12)60745-4

[8] EFE. Government does U-turn on healthcare access for immigrants. El Pais. 2015.

[9] Rechel B, Mladovsky P, Ingleby D, Mackenbach JP, McKee M. Migration and health in an increasingly diverse Europe. Lancet. 2013 4 6;381(9873):1235–45. 10.1016/S0140-6736(12)62086-8 23541058

[10] Davies AA, Basten A, Frattini C. Migration: A Social Determinant of the Health of Migrants. Brussels: International Organization for Migration (IOM), 2006.

[11] Requena-Mendez A, Aldasoro E, de Lazzari E, Sicuri E, Brown M, Moore DA, et al Prevalence of Chagas disease in Latin-American migrants living in Europe: a systematic review and meta-analysis. PLoS neglected tropical diseases. 2015 2;9(2):e0003540 Pubmed Central PMCID: 4332678. 10.1371/journal.pntd.0003540 25680190 PMC4332678

[12] Ingleby D. How health systems can address health inequities linked to migration and ethnicity Copenhagen: WHO Europe; 2010.

[13] WHO. Health for all policy framework for the WHO European Region: 2005 update. Geneva: WHO; 2005.

[14] Mladovsky P, Rechel B, Ingleby D, McKee M. Responding to diversity: an exploratory study of migrant health policies in Europe. Health policy. 2012 4;105(1):1–9. 10.1016/j.healthpol.2012.01.007 22306024

[15] García-Ramirez M, Hatzidimitriadou E. User involvement and empowerment in health care practices with ethnic minority and migrant groups: a community approach. "Editorial", International Journal of Migration, Health and Social Care. 2009; 5 (1): 2–4.

[16] Pope C, Mays N. Reaching the parts other methods cannot reach: an introduction to qualitative methods in health and health services research. Bmj. 1995 7 1;311(6996):42–5. . Pubmed Central PMCID: 2550091. 7613329 10.1136/bmj.311.6996.42 PMC2550091

[17] Pope C, Ziebland S, Mays N. Qualitative research in health care. Analysing qualitative data. Bmj. 2000 1 8;320(7227):114–6. . Pubmed Central PMCID: 1117368. 10625273 10.1136/bmj.320.7227.114 PMC1117368

[18] Dahlgren G, Whitehead M. Policies and Strategies to Promote Social Equity in Health. Stockholm: Institute for Futures Studies; 1991.

[19] QSR International. NVIVO 10 for windows. Available: http://www.qsrinternational.com/products_nvivo.aspx.

[20] Bazeley P, Jackson K. Qualitative Data Analysis with NVIVO. London: Sage; 2013.

[21] McDonald JT, Kennedy S. Insights into the 'healthy immigrant effect': health status and health service use of immigrants to Canada. Soc Sci Med. 2004 10;59(8):1613–27. . 15279920 10.1016/j.socscimed.2004.02.004

[22] Kunst A, Stronks K, Agyemang C. Non-Communicable diseases In: Rechel Bernd, Mladovsky Philipa, Walter Devillé, Barbara Rijks, Roumyana Petrova-Benedict, Martin McKee, editors. Migration and health in the EU. Berkshire: European Observatory on Health Systems and Policies Series; 2011 pp.101–120.

[23] Gill PS, Kai J, Bhopal RS, Wild S. Black and Minority Ethnic Groups In: Raftery J. Abingdon, editors. Health Care Needs Assessment. The epidemiologically based needs assessment reviews. Third Series, Abingdon: Radcliffe Medical Press Ltd; 2007 pp:227–399.

[24] Brunner E, Marmot M. Social organization, stress and health In: Marmot MG, Wilkinson R, editors. Social Determinants of Health. Oxford: Oxford University Press; 2011.

[25] Brunner E. Stress and the biology of inequality. Bmj. 1997 5 17;314(7092):1472–6. . Pubmed Central PMCID: 2126744. 9167568 10.1136/bmj.314.7092.1472 PMC2126744

[26] Kristenson M, Zieden B, Kucinskiene Z, Elinder LS, Bergdahl B, Elwing B, et al Antioxidant state and mortality from coronary heart disease in Lithuanian and Swedish men: concomitant cross sectional study of men aged 50. Bmj. 1997 3 1;314(7081):629–33. . Pubmed Central PMCID: 2126116. 9066473 10.1136/bmj.314.7081.629 PMC2126116

[27] Kristenson M, Eriksen HR, Sluiter JK, Starke D, Ursin H. Psychobiological mechanisms of socioeconomic differences in health. Social science & medicine. 2004 4;58(8):1511–22. . 14759694 10.1016/S0277-9536(03)00353-8

[28] McEwen BS, Stellar E. Stress and the individual. Mechanisms leading to disease. Archives of internal medicine. 1993 9 27;153(18):2093–101. . 8379800

[29] Factor R, Kawachi I, Williams DR. Understanding high-risk behavior among non-dominant minorities: a social resistance framework. Social science & medicine. 2011 11;73(9):1292–301. . 21907476 10.1016/j.socscimed.2011.07.027

[30] WHO Europe. Social determinants of health The solid facts. Copenhagen: WHO; 2003.

[31] Nyberg ST, Fransson EI, Heikkila K, Alfredsson L, Casini A, Clays E, et al Job strain and cardiovascular disease risk factors: meta-analysis of individual-participant data from 47,000 men and women. PloS one. 2013;8(6):e67323 Pubmed Central PMCID: 3688665. 10.1371/journal.pone.0067323 23840664 PMC3688665

[32] Kivimaki M, Leino-Arjas P, Luukkonen R, Riihimaki H, Vahtera J, Kirjonen J. Work stress and risk of cardiovascular mortality: prospective cohort study of industrial employees. Bmj. 2002 10 19;325(7369):857 . Pubmed Central PMCID: 129630. 12386034 10.1136/bmj.325.7369.857 PMC129630

[33] Siegrist Johannes. Effort-reward imbalance at work and health In: Pamela L. Perrewe, Daniel C. Ganster, editors. Historical and Current Perspectives on Stress and Health (Research in Occupational Stress and Well-being, Volume 2). Emerald Group Publishing Limited; 2002 pp.261–291.

[34] Bosma H, Peter R, Siegrist J, Marmot M. Two alternative job stress models and the risk of coronary heart disease. American journal of public health. 1998 1;88(1):68–74. . Pubmed Central PMCID: 1508386. 9584036 10.2105/ajph.88.1.68 PMC1508386

[35] Karasek R, Baker D, Marxer F, Ahlbom A, Theorell T. Job decision latitude, job demands, and cardiovascular disease: a prospective study of Swedish men. American journal of public health. 1981 7;71(7):694–705. . Pubmed Central PMCID: 1619770. 7246835 10.2105/ajph.71.7.694 PMC1619770

[36] Knutsson A, Hallquist J, Reuterwall C, Theorell T, Akerstedt T. Shiftwork and myocardial infarction: a case-control study. Occupational and environmental medicine. 1999 1;56(1):46–50. . Pubmed Central PMCID: 1757657. 10341746 10.1136/oem.56.1.46 PMC1757657

[37] Ferrie JE, Martikainen P, Shipley MJ, Marmot MG, Stansfeld SA, Smith GD. Employment status and health after privatisation in white collar civil servants: prospective cohort study. Bmj. 2001 3 17;322(7287):647–51. . Pubmed Central PMCID: 26544. 11250849 10.1136/bmj.322.7287.647 PMC26544

[38] Bartley M. Unemployment and ill health: understanding the relationship. J Epidemiol Community Health. 1994 8;48(4):333–7. . Pubmed Central PMCID: 1059979. 7964329 10.1136/jech.48.4.333 PMC1059979

[39] Ronda Perez E, Benavides FG, Levecque K, Love JG, Felt E, Van Rossem R. Differences in working conditions and employment arrangements among migrant and non-migrant workers in Europe. Ethnicity & health. 2012;17(6):563–77. . 23534504 10.1080/13557858.2012.730606

[40] Ronda E, Agudelo-Suarez AA, Garcia AM, Lopez-Jacob MJ, Ruiz-Frutos C, Benavides FG. Differences in exposure to occupational health risks in Spanish and foreign-born workers in Spain (ITSAL Project). Journal of immigrant and minority health/ Center for Minority Public Health. 2013 2;15(1):164–71. 10.1007/s10903-012-9664-9 22739799

[41] Ahonen EQ, Lopez-Jacob MJ, Vazquez ML, Porthe V, Gil-Gonzalez D, Garcia AM, et al Invisible work, unseen hazards: The health of women immigrant household service workers in Spain. American journal of industrial medicine. 2010 4;53(4):405–16. 10.1002/ajim.20710 19479889

[42] International Labour Office: Promoting integration for migrant domestic workers in Belgium-Executive Summary. Centre for Migration and Intercultural Studies (CeMIS), University of Antwerp; 2013.

[43] Marmot M, Allen J, Bell R, Bloomer E, Goldblatt P, Consortium for the European Review of Social Determinants of Health. WHO European review of social determinants of health and the health divide. Lancet. 2012 9 15;380(9846):1011–29. 10.1016/S0140-6736(12)61228-8 22964159

[44] Viruell-Fuentes EA. Beyond acculturation: immigration, discrimination, and health research among Mexicans in the United States. Social science & medicine. 2007 10;65(7):1524–35. . 17602812 10.1016/j.socscimed.2007.05.010

[45] Graham GG, Davies MJ, Day RO, Mohamudally A, Scott KF. The modern pharmacology of paracetamol: therapeutic actions, mechanism of action, metabolism, toxicity and recent pharmacological findings. Inflammopharmacology. 2013 6;21(3):201–32. 10.1007/s10787-013-0172-x 23719833

[46] Gutthann SP, Garcia Rodriguez LA, Raiford DS. Individual nonsteroidal antiinflammatory drugs and other risk factors for upper gastrointestinal bleeding and perforation. Epidemiology. 1997 1;8(1):18–24. . 9116088 10.1097/00001648-199701000-00003

[47] Lam JR, Schneider JL, Zhao W, Corley DA. Proton pump inhibitor and histamine 2 receptor antagonist use and vitamin B12 deficiency. Jama. 2013 12 11;310(22):2435–42. 10.1001/jama.2013.280490 24327038

[48] Roberts E, Delgado Nunes V, Buckner S, Latchem S, Constanti M, Miller P, et al Paracetamol: not as safe as we thought? A systematic literature review of observational studies. Annals of the rheumatic diseases. 2015 3 2 . 25732175 10.1136/annrheumdis-2014-206914 PMC4789700

[49] Williams CM, Maher CG, Latimer J, McLachlan AJ, Hancock MJ, Day RO, et al Efficacy of paracetamol for acute low-back pain: a double-blind, randomised controlled trial. Lancet. 2014 11 1;384(9954):1586–96. 10.1016/S0140-6736(14)60805-9 25064594

[50] Napier AD, Ancarno C, Butler B, Calabrese J, Chater A, Chatterjee H, et al Culture and health. Lancet. 2014 11 1;384(9954):1607–39. 10.1016/S0140-6736(14)61603-2 25443490

[51] Castañeda H. Immigration and health: conceptual, methodological, and theoretical propositions for applied anthropology NAPA Bulletin. 2010;34:6–27.

[52] Conrad P. The shifting engines of medicalization. Journal of health and social behavior. 2005 3;46(1):3–14. . 15869117 10.1177/002214650504600102

[53] Villarroel N, Artazcoz L. Heterogeneous patterns of health status among immigrants in Spain. Health & place. 2012 11;18(6):1282–91. . English. 23085201 10.1016/j.healthplace.2012.09.009

[54] Lindert J, Ehrenstein OS, Priebe S, Mielck A, Brahler E. Depression and anxiety in labor migrants and refugees-a systematic review and meta-analysis. Social science & medicine. 2009 7;69(2):246–57. . 19539414 10.1016/j.socscimed.2009.04.032

[55] Brigidi S. Culturas médicas y emigrantes en el casco antiguo de Génova In: Josep M.Comelles, Xavier Allué, Mariola Bernal, José Fernández-Rufete, Mascarella Laura, editors. Migraciones y Salud. Tarragona: Universitat Rovira i Virgili; 2009 pp 300–315.

[56] Espinosa P. Los Ulises que viven en Andalucía. El Pais 2013.

[57] Aragona M, Rovetta E, Pucci D, Spoto J, Villa AM. Somatization in a primary care service for immigrants. Ethnicity and Health. 2012 01 10;17(5):477–91. 10.1080/13557858.2012.661406 22352805

[58] Patino C, Kirchner T. Stress and psychopathology in latin-american immigrants: The role of coping strategies. Psychopathology. 2010 12;43(1):17–24. 10.1159/000255959 19893340

[59] Kleinman A, Benson P. Anthropology in the clinic: the problem of cultural competency and how to fix it. PLoS medicine. 2006 10;3(10):e294 . Pubmed Central PMCID: 1621088. 17076546 10.1371/journal.pmed.0030294 PMC1621088

[60] Lo MC, Stacey CL. Beyond cultural competency: Bourdieu, patients and clinical encounters. Sociology of health & illness. 2008 7;30(5):741–55. . 18444951 10.1111/j.1467-9566.2008.01091.x

[61] Kleinman A. Culture and Depression. Los Angeles, California: University of California Press; 1985

[62] Frank J. Book review: Social Perspective: The Missing Element in Mental Health by Richard U'Ren. University of Toronto Press, 2011. J Epidemiol Community Health. 2013;67(5):469–70.

[63] Cruz I, Serna C, Real J, Rue M, Soler J, Galvan L. Comparison of the consumption of antidepressants in the immigrant and native populations in a Spanish health region: an observational study. BMC public health. 2010;10:255 Pubmed Central PMCID: 2888739. 10.1186/1471-2458-10-255 20478063 PMC2888739

[64] Moynihan R, Heneghan C, Godlee F. Too much medicine: from evidence to action. Bmj. 2013;347:f7141 10.1136/bmj.f7141 24307723

[65] Glasziou P, Moynihan R, Richards T, Godlee F. Too much medicine; too little care. Bmj. 2013;347:f4247 10.1136/bmj.f4247 23820022

[66] Coyne JC, de Jonge P. Should African Americans be overtreated for depression the same as whites are? Commentary on Waldman et al (2009). American heart journal. 2009 5;157(5):e31; author reply e5-7. 10.1016/j.ahj.2009.03.004 19376297

[67] Ganann R. Opportunities and challenges associated with engaging immigrant women in participatory action research. Journal of immigrant and minority health / Center for Minority Public Health. 2013 4;15(2):341–9. 10.1007/s10903-012-9622-6 22491996

[68] Bonney R, Shirk JL, Phillips TB, Wiggins A, Ballard HL, Miller-Rushing AJ, et al Citizen science. Next steps for citizen science. Science. 2014 3 28;343(6178):1436–7. 10.1126/science.1251554 24675940

[69] Boulanger RF, Seidel S, Lessem E, Pyne-Mercier L, Williams SD, Mingote LR, et al Engaging communities in tuberculosis research. The Lancet Infectious diseases. 2013 6;13(6):540–5. 10.1016/S1473-3099(13)70042-2 23531390

[70] Horowitz CR, Robinson M, Seifer S. Community-based participatory research from the margin to the mainstream: are researchers prepared? Circulation. 2009 5 19;119(19):2633–42. Pubmed Central PMCID: 2796448. 10.1161/CIRCULATIONAHA.107.729863 19451365 PMC2796448

[71] Guta A, Flicker S, Roche B. Governing through community allegiance: a qualitative examination of peer research in community-based participatory research. Critical public health. 2013 12;23(4):432–51. . Pubmed Central PMCID: 3827674. 24273389 10.1080/09581596.2012.761675 PMC3827674

[72] Guta A, Strike C, Flicker S, Murray SJ, Upshur R, Myers T. Governing through community-based research: lessons from the Canadian HIV research sector. Social science & medicine. 2014 12;123:250–61. . 25074512 10.1016/j.socscimed.2014.07.028

