# Supplementary material for: "Carrying Ibuprofen in the Bag": Priority Health Concerns of Latin American Migrants in Spain- A Participatory Qualitative Study
Source: PLoS One. 2015 Aug 28;10(8):e0136315. doi: 10.1371/journal.pone.0136315 (PMC4552793; doi:10.1371/journal.pone.0136315)
Supplement: S1 File — (PDF) [file pone.0136315.s001.pdf]

Actividad piloto de investigación participativa con inmigrantes Latino Americanos en Barcelona

<u>Localización</u>
Tendrá lugar preferentemente en una sala donde el grupo se suela reunir

GUÍA DE FACILITACIÓN

Antes de que la sesión empiece deberá asegurarse de que todos los participantes están cómodos y pueden verse y escucharse los unos a los otros.

Parte I. Presentaciones y consentimiento informado
🕝 30 minutos

Agradece a los participantes que hayan venido. Preséntate a ti misma y a la persona que tomará las notas.

Explica a los participantes que esta actividad es parte del proyecto COHEMI el cual pretende ampliar el conocimiento que se tiene sobre la salud de los inmigrantes Latino Americanos en Europa.

Con esta actividad lo que pretendemos en concreto es poder entender mejor cuáles son los principales **PROBLEMAS DE SALUD** de los Latino Americanos en Europa, sus **CAUSAS**, y las posibles **BARRERAS** que puedan encontrar para ACCEDER a los servicios médicos.

Creemos que una buena manera de entender dónde se deben dirigir los esfuerzos de investigación es preguntarles a Uds. directamente. Esta actividad se realizará con diferentes grupos de inmigrantes Latino Americanos tanto aquí en Barcelona como en otras ciudades.

- Para nosotros es muy importante aprender sobre sus experiencias, preocupaciones y sugerencias. La razón por la que nos interesa aprender acerca de las dificultades y retos que afrontan en relación a su salud y el acceso a servicios sanitarios es que al identificar y priorizar los problemas podemos empezar a pensar en medidas para mejorar la situación.
- Aunque trataremos de crear conciencia, no estamos en posición de garantizar que podremos dar respuesta a las necesidades identificadas.
- Nuestras discusiones serán en formato de GRUPO. Aunque todo lo que digáis será mantenido en confidencialidad**, no os pedimos que compartáis experiencias personales que no deseéis compartir.**
- Se tomarán notas de las discusiones y se hará una grabación pero nunca se utilizarán vuestros nombres y nadie ajeno a este proyecto podrá asociar una determinada opinión a una persona en concreto. Todos los profesionales que participamos en este estudio tenemos un firme compromiso con el tratamiento ABSOLUTAMENTE CONFIDENCIAL de la información que nos facilitéis.
- Si en algún momento no estáis cómodos con la conversación o las actividades realizadas, por favor sentiros libres de abandonar la actividad. Tenéis ABSOLUTA LIBERTAD para dejar la sala cuando lo deseéis o no participar en algún ejercicio, si así lo deseáis.
- Si tenéis cualquier duda os la podemos aclara ahora.

Si en cualquier otro momento os surgiera alguna pregunta adicional sentiros libres de contactarme en el teléfono: XXXXX (no personal!)

- **Están todos de acuerdo en proseguir con esta actividad?**

Nota: Con anterioridad a la actividad ya se habrá dado información a los participantes acerca del carácter grupal de la actividad, ya que para algunas personas puede ser difícil negarse a participar una vez el grupo ya se ha reunido

PRESENTACIONES

Explica a los participantes que para empezar nos gustaría que se presentaran eligiendo un "mote" - que será el utilizado para tomar las notas- y algo que les gusta hacer. El "mote" se lo debe prender cada participante con una nota grande en la ropa y puede ser el nombre de alguien que admiren o les guste mucho. Pide a los participantes que se vayan levantando en turnos presentándose a los demás diciendo por ejemplo: *Mi mote es XX y me gusta bailar. A quien le guste bailar, que se levante! (o arriba!)*

Parte II. **NUESTRA SALUD**
🕐 **2 horas**

Objetivo: Identificar cuales son los **PROBLEMAS DE SALUD PERCIBIDOS COMO PRIORITARIOS**

Actividad: "lluvia de ideas" y ejercicio de priorización (ranking)

Una vez los participantes se han relajado, explica que ahora vamos a realizar una actividad pensando en un personaje hipotético. Sitúa la silueta de una mujer en la pared y pide a los participantes que le den un nombre que se escribirá al lado de la silueta para referirse a ella durante toda la actividad.

Pregúntale al grupo que piense sobre **TODOS LOS POSIBLES PROBLEMAS DE SALUD QUE PUEDE HABER TENIDO DESDE QUE LLEGÓ.** Estimula una "lluvia de ideas" pero no intervengas hasta que dejen de hablar → Sólo entonces, explica que deben pensar de forma abierta, pensando en muchos tipos diferentes de problemas de salud (graves, menos graves, físicos, mentales, crónico, pasajeros, causados por virus, animales, condiciones de vida…)

Se deberá diferenciar claramente en las "notas de campo" entre los problemas que surgen espontáneamente del grupo y aquellos que surgen tras la intervención del facilitador/a.

→ Cuando no mencionen nada más, pregunta: ¿podéis pensar en algún otro problema? ¿Puede alguien mencionar otro problema de salud que pueda haber tenido?

Mientras tanto se van tomado notas de todos los problemas mencionados que se escribirán en tarjetas individuales de tamaño DNA 4.

Explica que cada una de las tarjetas representa un "ladrillo" y que vamos a construir el MURO que le impide gozar de buena salud.

EL MURO

Se representará una línea en una de las paredes de la sala para hacer un "ranking" de los problemas de salud identificados.

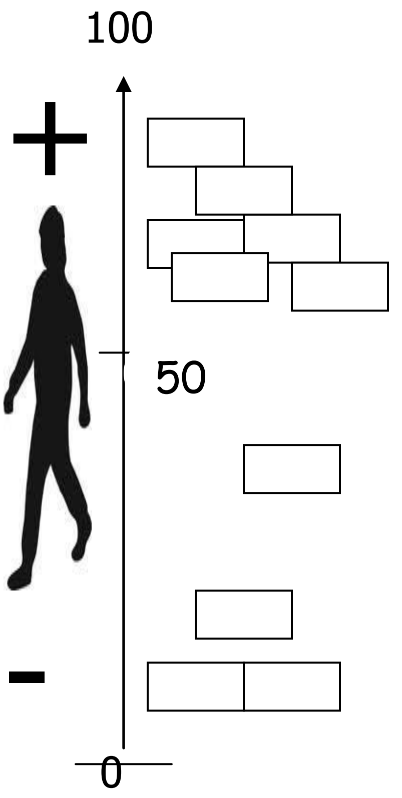

Explica a los participantes que ahora queremos ver qué alto es el muro que le impide gozar de buena salud. Explica que cada tarjeta representa un ladrillo, y que todos juntos formaran su estado de salud. Queremos saber cuáles son los problemas más importantes, que situaremos en la parte de arriba del muro. Los menos relevantes se situarán en la parte inferior.

→ Empieza por coger una tarjeta al azar y pregúntales: ¿donde ponemos este problema? **¿Es un problema muy grande y lo ponemos arriba? ¿No es tan importante y lo debemos poner por el medio? ¿Es poco importante y debe ir abajo?**

Estimula una discusión sobre **POR QUÉ** ese problema es tan- o no tan-importante. Cuando dejen de hablar, estimula el debate: ¿Qué es lo que hace que un problema se perciba como importante **¿frecuencia? ¿dolor? ¿incapacidad para trabajar? ¿otros?** Ayuda a los participantes a consensuar donde poner el ladrillo: ¿están todos Uds. de acuerdo? ¿Por qué si? ¿Porque no? (no importa si no hay consenso. Lo importante es la discusión generada en el proceso). Repite el proceso para las diferentes tarjetas estimulando discusión acerca de por qué unos problemas se perciben como más importantes que otros. Cambia la posición de las tarjetas si es necesario.
¿Sería el muro diferente si fuera más joven? ¿y si fuera más mayor?¿si fuera un hombre?

¿POR QUÉ?

Explica a los participantes que ahora queremos saber cuáles son las **CAUSAS** de los problemas de salud que tiene. Recorre el muro, y para cada tarjeta pregunta *¿cuáles son las posibles causas de este problema?*

Estimula la lluvia de ideas: *¿algo más? ¿podéis pensar en alguna causa adicional?*

Cuando dejen de hablar, estimula la discusión sobre un amplio abanico de causas posibles incluyendo aspectos relacionados con las condiciones de vida, vivienda, trabajo…

Parte III **UTILIZACIÓN DE SERVICIOS SANITARIOS**
🕐 **1 hora**

Explica a los participantes que ahora queremos saber **QUÉ HACE CUANDO TIENE UN PROBLEMA DE SALUD**. Pide a los participantes que escojan un problema de salud del muro. Estimula la discusión sobre por qué han elegido ese problema en particular.

Pide a los participantes que piensen en QUÉ HARÍAN para solucionar ese problema **¿Qué medidas tomarían? ¿A quien consultarían? ¿Qué remedios utilizarían?**

¿Desearían ir a un centro de salud? ¿Por qué si? ¿Por qué no?

Sitúa una línea horizontal en una de las paredes de la sala y explica a los participantes que ahora vamos a suponer que desean ir a un centro de salud para solucionar su problema. Queremos ver qué obstáculos podría encontrar.

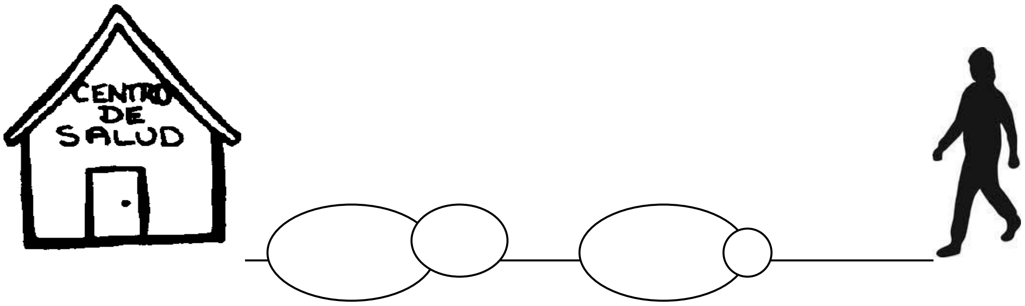

Pide a los participantes que piensen en todas las posibles causas que podrían hacer difícil o imposible llegar a recibir la atención deseada.

En esta ocasión el ejercicio será individual. Cada participante tendrá tres fichas redondeadas de diferentes tamaños que representarán las "piedras" u obstáculos para acceder a los servicios.

Si es necesario, enfatiza que será necesario pensar en un amplio abanico de factores incluyendo aspectos psicológicos y sociales, para ir más allá de dificultades relacionadas con el coste y el viaje. Cuando cada participante termine, las fichas se entregarán al facilitador que hará una exposición de los temas que se mencionan, situándolos a lo largo del camino y estimulando discusión sobre los temas considerados de mayor relevancia.

Parte IV. **ÁRBOL DE SOLUCIONES**
🕐 **2 horas**

<u>Objetivo</u>: identificar posibles soluciones para mitigar las barreras de acceso a servicios identificados en la actividad anterior **sin generar falsas expectativas**.

Explica que ahora vamos a pensar juntos sobre qué se podría hacer para ayudar a superar los obstáculos. Divide los participantes en grupos de 3-4 persona. Distribuye a cada grupo una de las tarjetas representando "piedras" grandes y un set de 4 tarjetas representando la forma de una fruta. Explica que ahora se trata de pensar sobre posibles soluciones para los problemas representados por las "piedras". Para cada "piedra" los participantes deberán considerar:

¿Cómo se podría eliminar o reducir este obstáculo? ¿Qué se puede hacer en la práctica? ¿QUIÉN lo puede hacer?

→ Estimula que piensen en todas las posibles personas involucradas, no solo los profesionales sanitarios y los políticos.

¿Qué se puede hacer desde las comunidades? ¿Y las familias? ¿Las parejas? ¿Los grupos religiosos, asociaciones juveniles y deportivas, grupos de baile, líderes comunitarios? ¿ALGUIEN MÁS?

Pide a cada uno de los sub-grupos que escriban en cada "fruta" posibles soluciones a las barreras representadas por las "piedras". Anímales a pensar sobre cuáles de las soluciones son más o menos factibles/realistas/alcanzables. Tras media hora, pide a un representante de cada grupo que resuma al resto de participantes las posibles soluciones en las que han pensado.
Se habrá situado una figura representando un árbol en una de las paredes de la sala.

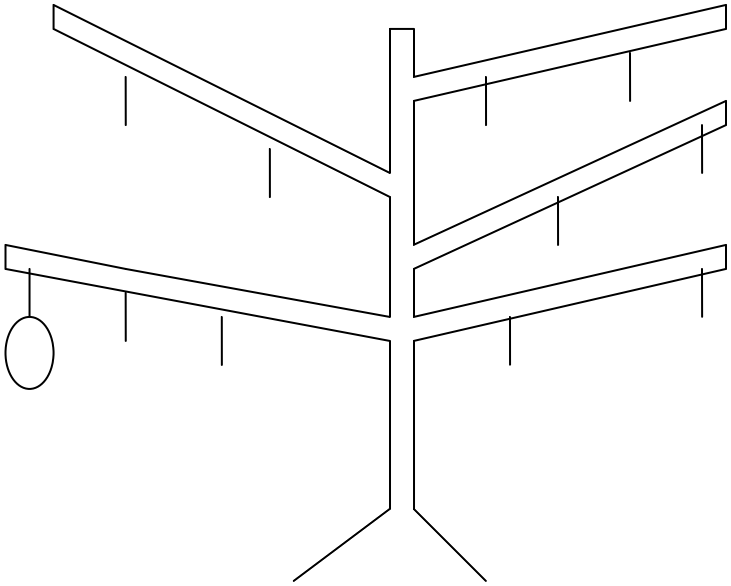

Los representantes de cada sub-grupo tendrán que colgar las Frutas representando soluciones en las ramas del árbol. Las "soluciones" más fáciles de obtener se situarán en las ramas bajas, mientras que las más difíciles se situarán en las ramas superiores. Una vez se hayan colgado todas las frutas, se estimulara la discusión sobre porqué unas "frutas" se ha localizado más arriba y otras más abajo. **¿Están todos de acuerdo? ¿Se debería cambiar la posición de alguno de los "frutos"?** Re-coloca las tarjetas según sea necesario siguiendo las discusiones y acuerdos del grupo y evita la creación de falsas expectativas acerca de posibles soluciones difíciles de implementar en la práctica.

Para CONCLUIR

☑ Haz una recopilación de lo que se ha discutido en la actividad
☑ Responde a las preguntas que puedan surgir
☑ Pregunta qué les ha parecido la actividad y si les gustaría participar en otras similares.
☑ Cuando hayan salido los participantes, ampliar las notas a partir de la información compilada en las diferentes paredes y tomar fotos de todos los ejercicios realizados por el grupo
